# Supplementary material for: Length of stay to recover from severe acute malnutrition and associated factors among under-five years children admitted to public hospitals in Aksum, Ethiopia
Source: PLoS One. 2020 Sep 29;15(9):e0238311. doi: 10.1371/journal.pone.0238311 (PMC7523984; doi:10.1371/journal.pone.0238311)
Supplement: Attachment — Submitted filename: PONE-D-20-03753_reviewer.pdf [file pone.0238311.s002.pdf]

PLOS ONE

Length of stay to recover from severe acute malnutrition and associated factors among under-five years children admitted to public Hospitals in Aksum, Ethiopia

--Manuscript Draft--

Manuscript Number: PONE-D-20-03753

Article Type: Research Article

Full Title: Length of stay to recover from severe acute malnutrition and associated factors among under-five years children admitted to public Hospitals in Aksum, Ethiopia

Short Title: Length of stay to recover from severe acute malnutrition and associated factors among under-5 years children

Corresponding Author: Tekia Zafu
Aksum University
Axum, Tigrai ETHIOPIA

Keywords: length of stay, severe acute malnutrition, children, hospital, Aksum

Abstract:

Background: Severe acute malnutrition is defined by <70% weight for length/height, by visible severe wasting, by the presence of pitting edema, and in children 6 to 59 months of age, mid upper arm circumference <110 mm (1–3). Severe acute malnutrition remains to be a worldwide problem, claiming lives of millio[illegible] especially in sub-Saharan Africa and south Asia. Though the Ethiopian [illegible] guideline states the total length of stay in therapeutic feeding units sho[illegible] than four weeks, there is huge difference, varying from 8 to 47 days of [illegible] objective of this study was to assess length of stay to recover from sev[illegible] malnutrition and associated factors among under five children hospitalized to the public hospitals in Aksum from September 11, 2016 to February 10, 2019.

this is not necessary to mention here. push tis to methodology

Methods: Sample size was calculated using STATA version 12.0 and retrospective cohort study was conducted using pretested questionnaire in the public hospitals in Aksum on children aged 0-59 months. Cleaned data was entered to Epi info version 7.1.4 and then exported into SPSS version 21 for analysis. Biva[illegible] analysis was performed using Kaplan Meier and Cox regressio[illegible] analysis, those with p-value < 0.05 were selected for multivariable analysis using Cox regression model to identify factors associated with length of stay.

were

presentation

Results: A total of 564 participants were enrolled to the study. The rate of recovery was 56% with median length of stay of 15 days (95% CI: 14.1, 15.9). The independent predictors of length of stay till recovery were presenting with diarrhea (AHR=0.573, 95% CI: 0.415-0.793), reactive HIV test (AHR=0.391, 95% CI: 0.194-0.788), palmar pallor (AHR=0.575, 95% CI: 0.416-0.794), co-morbidity (AHR=0.415, 95% CI: 0.302-0.570) at admission and not being treated with plumpy nut (AHR=0.368, 95% CI: 0.262-0.518), not blood transfused (AHR=1.905, 95% CI: 1.158-3.135), no IV fluid resuscitation (AHR=1.548, 95% CI: 1.074-2.232) and not fed with NG tube (AHR=1.861, 95% CI: 1.335-2.593).

Conclusions: Length of stay is in the acceptable range of the international and national set of standards. Nevertheless, the cure rate was lower compared to the Sphere standards. Presence of co-morbidity, diarrhea, pallor and HIV, and not receiving plumpy nut should be prevented while not being transfused, not being infused and not fed with naso-gastric tube were protective for early recovery.

Key words: length of stay, severe acute malnutrition, children, hospital, Aksum.

Order of Authors:
Wagnew Tesfay
Tekia Zafu
Mebrahtu Abay
Solomon Hintsa

Additional Information:

Question	Response
Financial Disclosure	The authors received no specific funding for this work

Enter a financial disclosure statement that describes the sources of funding for the work included in this submission. Review the submission guidelines for detailed requirements. View published research articles from *PLOS ONE* for specific examples.<br><br>This statement is required for submission and **will appear in the published article** if the submission is accepted. Please make sure it is accurate.<br><br>**Unfunded studies**<br>Enter: *The author(s) received no specific funding for this work.*<br><br>**Funded studies**<br>Enter a statement with the following details:<br>• Initials of the authors who received each award<br>• Grant numbers awarded to each author<br>• The full name of each funder<br>• URL of each funder website<br>• Did the sponsors or funders play any role in the study design, data collection and analysis, decision to publish, or preparation of the manuscript?<br>• **NO** - Include this sentence at the end of your statement: *The funders had no role in study design, data collection and analysis, decision to publish, or preparation of the manuscript.*<br>• **YES** - Specify the role(s) played.<br><br>* typeset	
**Competing Interests**<br><br>Use the instructions below to enter a competing interest statement for this submission. On behalf of all authors, disclose any competing interests that could be perceived to bias this work—acknowledging all financial support and any other relevant financial or non-financial competing interests.<br><br>This statement **will appear in the published article** if the submission is accepted. Please make sure it is accurate. View published research articles	The authors have declared that no competing interests exist.

from *PLOS ONE* for specific examples.<br><br>**NO authors have competing interests**<br><br>Enter: *The authors have declared that no competing interests exist.*<br><br>**Authors with competing interests**<br><br>Enter competing interest details beginning with this statement:<br><br>*I have read the journal's policy and the authors of this manuscript have the following competing interests: [insert competing interests here]*<br><br>* typeset	
**Ethics Statement**<br><br>Enter an ethics statement for this submission. This statement is required if the study involved:<br><br>• Human participants<br>• Human specimens or tissue<br>• Vertebrate animals or cephalopods<br>• Vertebrate embryos or tissues<br>• Field research<br><br>Write "N/A" if the submission does not require an ethics statement.<br><br>General guidance is provided below. Consult the submission guidelines for detailed instructions. **Make sure that all information entered here is included in the Methods section of the manuscript.**	Ethical clearance and permission letter were taken from the Ethical Review Committee (ERC) of Aksum University, College of health science. An official letter was written to Selekleka primary hospital requesting facilitation to conduct the research study and this official letter approved and distributed to the respective units of Selekleka primary hospital. Confidentiality and privacy was maintained during data collection, analysis and reporting in which the information obtained from the data will not be shared other than the data collectors and principal investigator and this was assured by obtaining institutional written consent from Selekleka primary hospital and by providing Information sheet which explains the purpose, benefit, and short and long-term effect of the study on the study participants.

Powered by Editorial Manager® and ProduXion Manager® from Aries Systems Corporation

**Format for specific study types**<br><br>**Human Subject Research (involving human participants and/or tissue)**<br>• Give the name of the institutional review board or ethics committee that approved the study<br>• Include the approval number and/or a statement indicating approval of this research<br>• Indicate the form of consent obtained (written/oral) or the reason that consent was not obtained (e.g. the data were analyzed anonymously)<br><br>**Animal Research (involving vertebrate animals, embryos or tissues)**<br>• Provide the name of the Institutional Animal Care and Use Committee (IACUC) or other relevant ethics board that reviewed the study protocol, and indicate whether they approved this research or granted a formal waiver of ethical approval<br>• Include an approval number if one was obtained<br>• If the study involved *non-human primates*, add *additional details* about animal welfare and steps taken to ameliorate suffering<br>• If anesthesia, euthanasia, or any kind of animal sacrifice is part of the study, include briefly which substances and/or methods were applied<br><br>**Field Research**<br><br>Include the following details if this study involves the collection of plant, animal, or other materials from a natural setting:<br>• Field permit number<br>• Name of the institution or relevant body that granted permission	
**Data Availability**<br><br>Authors are required to make all data underlying the findings described fully available, without restriction, and from the time of publication. PLOS allows rare exceptions to address legal and ethical concerns. See the PLOS Data Policy and FAQ for detailed information.	Yes - all data are fully available without restriction

A Data Availability Statement describing where the data can be found is required at submission. Your answers to this question constitute the Data Availability Statement and **will be published in the article**, if accepted.<br><br>**Important:** Stating 'data available on request from the author' is not sufficient. If your data are only available upon request, select 'No' for the first question and explain your exceptional situation in the text box.<br><br>Do the authors confirm that all data underlying the findings described in their manuscript are fully available without restriction?	
**Describe where the data may be found in full sentences. If you are copying our sample text, replace any instances of XXX with the appropriate details.**<br><br>• If the data are **held or will be held in a public repository**, include URLs, accession numbers or DOIs. If this information will only be available after acceptance, indicate this by ticking the box below. For example: *All XXX files are available from the XXX database (accession number(s) XXX, XXX.).*<br>• If the data are all contained **within the manuscript and/or Supporting Information files**, enter the following: *All relevant data are within the manuscript and its Supporting Information files.*<br>• If neither of these applies but you are able to provide **details of access elsewhere**, with or without limitations, please do so. For example:<br><br>*Data cannot be shared publicly because of [XXX]. Data are available from the XXX Institutional Data Access / Ethics Committee (contact via XXX) for researchers who meet the criteria for access to confidential data.*<br><br>*The data underlying the results presented in the study are available from (include the name of the third party*	All relevant data will present up on request.

and contact information or URL).<br>• This text is appropriate if the data are owned by a third party and authors do not have permission to share the data.<br><br>* typeset	
Additional data availability information:	

Manuscript
Click here to access/download;Manuscript;Length of stay to recover.docx

Length of stay to recover from severe acute malnutrition and associated factors among under-five years children admitted to public Hospitals in Aksum, Ethiopia

Wagnew Tesfay [1], Mebrahtu Abay[2], Solomon Hintsa[3], Tekia Zafu[4*]

[1]Medecins Sans Frontieres Holland, Ethiopia mssion, Tigray project, Email: wagnew.tesfaye@gmail.com

[2]Department of Epidemiology and Biostatistics, School of Public Health, College of Health Science, Aksum University, Aksum, Ethiopia, Email: gmebrahtuabay@gmail.com

[3]Department of Epidemiology and Biostatistics, School of Public Health, College of Health Science, Aksum University, Aksum, Ethiopia, Email: solomonhintsa2013@gmail.com

[1]Department of Epidemiology and Biostatistics, School of Public Health, College of Health Science, Aksum University, Aksum, Ethiopia, Email: tekish2014@gmail.com

***Corresponding author**: Email: tekish2014@gmail.com, Tel: +251920256201

Abstract

Background: Severe acute malnutrition is defined by <70% weight for length/height, by visible severe wasting, by the presence of pitting edema, and in children 6 to 59 months of age, mid upper arm circumference <110 mm (1–3). Severe acute malnutrition remains to be a worldwide problem, claiming lives of millions of children, especially in sub-Saharan Africa and south Asia. Though the Ethiopian national guideline states the total length of stay in therapeutic feeding units should not be more than four weeks, there is huge difference, varying from 8 to 47 days of stay. So objective of this study was to assess length of stay to recover from severe acute malnutrition and associated factors among under five children hospitalized to the public hospitals in Aksum from September 11, 2016 to February 10, 2019.

Methods: Sample size was calculated using STATA version 12.0 and retrospective cohort study was conducted using pretested questionnaire in the public hospitals in Aksum on children aged 0-59 months. Cleaned data was entered to Epi info version 7.1.4 and then exported into SPSS version 21 for analysis. Bivariate and multivariate analysis was performed using Kaplan Meier and Cox regression. During bivariate analysis, those with p-value < 0.05 were selected for multivariable analysis using Cox regression model to identify factors associated with length of stay.

Results: A total of 564 participants were enrolled to the study. The rate of recovery was 56% with median length of stay of 15 days (95% CI: 14.1, 15.9). The independent predictors of length of stay till recovery were presenting with diarrhea (AHR=0.573, 95% CI: 0.415-0.793), reactive HIV test (AHR=0.391, 95% CI: 0.194-0.788), palmar pallor (AHR=0.575, 95% CI: 0.416-0.794), co-morbidity (AHR=0.415, 95% CI: 0.302-0.570) at admission and

not being treated with plumpy nut (AHR=0.368, 95% CI: 0.262-0.518), not blood transfused (AHR=1.905, 95% CI: 1.158-3.135), no IV fluid resuscitation (AHR=1.548, 95% CI: 1.074-2.232) and not fed with NG tube (AHR=1.861, 95% CI: 1.335-2.593).

Conclusions: Length of stay is in the acceptable range of the international and national set of standards. Nevertheless, the cure rate was lower compared to the Sphere standards. Presence of co-morbidity, diarrhea, pallor and HIV, and not receiving plumpy nut should be prevented while not being transfused, not being infused and not fed with naso-gastric tube were protective for early recovery.

Key words: length of stay, severe acute malnutrition, children, hospital, Aksum.

Introduction

Severe acute malnutrition (SAM) is defined by <70% weight for length/height (WFL/H), by visible severe wasting, by the presence of pitting edema, and in children 6 to 59 months of age, mid upper arm circumference (MUAC) <110 mm (1–3). Although there are basic and underlying causes, SAM is the immediate effect of inadequate dietary intake (quality or quantity) &/or infections like tuberculosis (TB), human immune deficiency virus/acquired immune deficiency syndrome (HIV/AIDS) and diarrhea that often lead to nutrient mal absorptions (4).

Although SAM usually affects all segments of a population, infants and young children are most vulnerable as they have higher nutritional requirements for growth and development (5). It is one of the leading causes of morbidity and mortality among infants and young children all over the world and more frequently in sub-Saharan Africa and south Asia (6).The peak age for SAM is 6-18 months, time of especially high growth velocity and brain

development. However, it is increasingly becoming common that SAM may occur in infants less than six months of age with many disadvantaged populations introducing solids to children as young as two months (3).

The 2013 Ethiopian guideline for SAM management states that the total length of stay (LOS) in therapeutic feeding units (TFUs) should not be more than four weeks (7). But there is huge difference in LOS in studies done in Ethiopia, varying from 8 to 47 days (8–10).

Severe acute malnutrition is a worldwide problem and one of the top deadly diseases for children less than five years of age. Severely malnourished children have a nine times more mortality rates than well-nourished children (11). As United nations' international children's emergency fund/world health organization/World bank stated in their joint report in 2018 (12), wasting threatened lives of an estimated 50.5 million children under five globally, of which 13.8 million are from Africa. Also there are 52 million (close to 8.3 per cent) children less than five suffering from acute malnutrition; out of those affected more than 90 per cent are from South & Southeast Asia & sub-Saharan Africa (13).

More than ten million children die before celebrating their fifth birthday each year globally due to SAM. Huge number of severely malnourished children die at their home without any hospital care, but sometimes even hospital care is given, death rates may be high (7). In developing countries, children under five who are severely malnourished and admitted to hospital face a 30 to 50 per cent case fatality rate, which is unacceptably high (14).

In the developing world, where SAM is the most common reason for pediatric hospitalization, it is associated with higher risk of morbidity & mortality, underlying for more than 50 per cent of the ten to eleven million children under 5 years old who die every

year from avoidable causes (2,15,16). Despite of such worldwide significance, child recovery programs have not given the required attention for facility based management of SAM (2).

According to studies from Kenya and Niger, recovery rate and LOS are affected by co-morbidities like pneumonia, malaria, altered consciousness, weak pulse, inability to drink, temperature gradient, chest in-drawing, diarrhea & severe pallor (17,18).

Horn of Africa has identified 6.6 per cent of children less than five years old as wasted. With estimated 10%, Ethiopia has highest rate of wasting (acute malnutrition) in this region (19). Even though it was not well known in this particular study area, some studies in Ethiopia indicated that there is a big variation in LOS in Ethiopia, ranging from 8 to 47 days (8–10), that is not congruent with the National SAM management protocol that states the total LOS in TFUs should not be more than four weeks (7). A study in northwest Ethiopia also revealed that patients admitted with SAM had a case fatality and defaulter rates of 18%, and 9% respectively (20).

UNICEF has conducted an evaluation study in Ethiopia (21) and identified gaps and management errors in the services like not giving routine medicines such as Amoxicillin, children who should be managed in phase two were managed in phase one, and those who could be treated in OTP were being treated in TFU, poor medical record handling, late transfer of cases from phase to phase & to discharge, and limited area allowed to the TFU that increase cross infection and raised risk of death, especially for HIV infected children as.

Though the Federal Ministry of Health (FMoH) jointly with its partners is taking promising actions in reducing SAM, the Ethiopian national underweight and wasting rates among under

five children are still high, 24 and 10 % respectively. The same is true in Tigray region as the underweight & wasting rates are 23 and 11% respectively (22,23).

There were couples of researches conducted on malnutrition in the study area, but none of them attempted to assess the length of stay to recover from SAM and associated factors among under five children hospitalized to public hospitals in Aksum town, northern Ethiopia. So, this research was intended to come up with information about length of stay till recovery and associated factors among severely malnourished children admitted to the identified health facilities for the betterment of quality of care.

Using the effective treatment protocols available for proper treatment of SAM plays a crucial role in achieving global child health targets. Though community based management of acute malnutrition (CMAM) is being advocated since recent years (7), health institution based treatment of SAM is still needed and particularly total length of stay until children admitted with SAM get recovered demands attention.

The purpose of this study was to assess the length of stay to recover from SAM and associated factors among under five children admitted to the public hospitals in Aksum, northern Ethiopia. Although there are couples of researches done on malnutrition in the mentioned study area, the proposed title and design was not addressed.

Do not repeat the objective

Moreover, coming up with recent information about length of stay till recovery and associated factors among severely malnourished children is enormously relevant to be used as input by clinicians for betterment of quality of care given to clients and as a guide (reference) for academicians who are interested to conduct further related research on the area. Therefore, the objective of this study was to assess length of stay to recover from SAM

and associated factors among children aged 0-59 months admitted to the public hospitals in Aksum town from September 11, 2016 to February 10, 2019, central zone of Tigray regional state, northern Ethiopia; 2019.

Methods and materials

Study area and period

Aksum is found in Tigray regional state, 1042 km far from the capital city of Ethiopia, Addis Ababa. St. Mary general hospital and Aksum University comprehensive specialized hospital are providing inpatient and outpatient services for approximately 2.8 million catchment population. Medical and health officer (HO) interns, nurses, general practitioners (GPs) and a pediatrician in each hospital are providing medical care and treatment for SAM children admitted to both health institutions. Admission, management and discharge procedures for SAM are compatible with the Protocol developed by Federal Ministry of Health (FMoH), that is updated in 2013 (7).

The study was carried out on those admitted with SAM from September 11, 2016 to February 10, 2019. Data was abstracted within 15 days (from March 6-20, 2019).

Study design

Health facility based retrospective cohort study was conducted in the two public hospitals (St. Mary and Aksum University comprehensive specialized hospital).

Source and study population

The source populations were all severely malnourished children admitted to TFUs of the public hospitals in Aksum. And the study populations were all severely malnourished children admitted to TFUs of the public hospitals in Aksum from September 11, 2016 to February 10, 2019.

Inclusion and exclusion criteria

Inclusion criteria

All severely malnourished under five children admitted to TFUs of the stated hospitals from September 11, 2016 to February 10, 2019, fulfilling the admission criteria set by FMoH guideline. Admission criteria, according to the National protocol for SAM management (7), are as follows;

Infants < 6 months or <3kg can be admitted to the NRUs when they have WFL <70% of median, presence of bilateral pitting edema, or visible severe wasting. Children aged 6-59 months can be admitted if they come up with WFL/WFH <70% of median, bilateral pitting edema, or MUAC <11Cm.

Exclusion criteria

This study has excluded children whose admission and discharge date not recorded as this will not show the outcome variable (LOS), those with congenital anomalies like cleft lip and developmental disorders such as Down's syndrome as these can be a confounding with LOS for SAM and those whose medical records were not found or incomplete for type of SAM.

Sample size and sampling procedure

Sample size was calculated based on sample size estimation for survival under the Cox proportional hazards model using the STATA Version 12.0, taking the following assumptions into account: a 1.8 adjusted hazard ratio, 17.1% (0.171) observed prevalence of failure/non-recovery (35), with marginal error of 5% and confidence interval of 95%. With the assumption of number of study subjects required to achieve a study power of 80% and 10% incompleteness, the overall sample become 585 **(Table 1).**

Study participants

All selected children aged 0-59 months with severe acute malnutrition admitted to NRUs of St. Mary general and Aksum University comprehensive specialized hospitals from September 11, 2016 to February 10, 2019 with clear inclusion and exclusion criteria.

The actual number of under five children admitted with SAM to the stated hospitals in the study period was estimated to be 700 (280 from Aksum University comprehensive specialized hospital and 420 from St. Mary hospital). Systematic random sampling technique was used to select the 585 study participants from the actual 700 taking each participant consecutively (700/585 = 1.2). With this proportion, 234 participants were selected consecutively from Aksum University comprehensive specialized hospital (AkUCSH) and 351 from St. Mary General hospital. Out of these eligible study participants, 564 of them were enrolled into the study whereas the remaining 21 (3.6%) children were excluded. Among these excluded, the clinical files for 12 children from St. Mary hospital were not found and one participant was removed during analysis as it was influential extreme value from same institution whereas total of eight children from AkUCSH were excluded (four

were admitted with congenital anomalies and developmental disorders (one with cleft lip and three with Down's syndrome) and clinical files were incomplete for type of SAM for the remaining four).

Variables of the study

Dependent/outcome variable:

Time till recovery (LOS)

Event: recovery

Censored: Not recovered from SAM. (Death, defaulter, medical referral, transferred)

Independent variables:

- Main exposure variable:

  Severe acute malnutrition diagnosis (marasmus, kwashiorkor marasmic-kwashiorkor)

  - Exposed: those with edematous type of SAM (kwashiorkor/marasmic-kwash)
  - Non-exposed: those with non-edematous type of SAM (marasmus)
- Other independent variables
  - Study subject characteristics (age, sex, residence)
  - Routine medications, supplements and therapeutic feedings
  - Co-morbidities (like HIV, malaria, TB, severe anemia)

Operational definitions

Length of stay (LOS): refers to the number of days/weeks it takes from hospitalization till when a child recovered from SAM of any kind. Children are called recovered when they got

relieved from medical complications, edema and have gained and maintained WFL/WFH of 85% (7).

Defaulters: those who were not found in the NRU for two successive days, or who leave the ward against professional advice while the child is not cured (7,13,32).

Death: when the SAM child die while receiving treatment in the TFU and registered as dead in the treatment logbook (7).

Data collection tools and data quality control

Data collection tools

A compilation sheet (coding check list) was developed relating to the FMoH standard management protocol for SAM. Then needed individual data was extracted from relevant documents like SAM registration logbook, SAM monitoring multi-chart and patient clinical files.

Data quality control

Compilation sheet (check list) was prepared in English that was pre-tested in Sehul general hospital in Shire using 5% of the total sample (29 participants) and revised for sequence and layout. Four Bachelor of Science degree holder health care professionals were recruited as data collectors with one supervisor. They got one day training on how to fill the check list to minimize errors. The collected data were checked by the principal investigator closely during data collection if accurate, complete & consistent. Corrective measures were taken after discussion with all the research team members and problems solved at the spot.

Then collected data were cleaned, coded and entered to Epi info version 7.1.4.0 and then exported to SPSS version 21 for analysis. The levels of missing values, existence of influential extreme values and multi co-linearity among independent variables was checked before analysis.

Data processing and analysis

The descriptive analysis was performed and presented in tables. Kaplan-Meier & Cox regression was applied to determine the association of independent variable with dependent. P-value of < 0.05 was taken as statistically significant to identify explanatory factors of LOS in multivariable analysis.

Model diagnostic procedures

Thirty one independent variables were analyzed in the Kaplan Meier bivariate analysis, out of which twenty one were suggestive of significance (p-value < 0.05) and entered to multivariable analysis using Cox proportional hazard regression. Stepwise forward likelihood ratio variable selection method with entry and removal probabilities of 0.05 and 0.1 respectively was made on significant factors in the bivariate analysis. Accordingly, the final Cox Proportional Hazards Model was fitted on the basis of final step selected variables after model assumption diagnostic procedures. Remedial measures were taken on potential problems found in checking validity of assumptions. In due course, neither multicollinearity problem nor significant interaction was detected. However, examination of Dfbeta statistics in relation to model coefficients indicated the existence of influential extreme value. Thus, the outlier was found to be the case entry with unique SAM serial number 411 and its removal resulted in magnitudes of 0.16 and 0.26 change to the corresponding model

coefficients of HIV Status (Table 6 in the Annex). As a result, the final model was fitted on 564 observations of selected variables.

The proportionality of hazards assumption was further checked by examining plots of recovery time for model variables. Patterns of the plot were not seemed to cross each other, rather were close to be parallel. This left no doubt about violation of proportionality of hazards assumption. Further this was ascertained by examination of cumulative hazard plot of the Cox-Snell residual. The plotted points nearly lie around a line that has unit slope and zero intercept, which also confirmed validity of proportional hazards assumption. Finally, goodness of the overall fit of the model was assured by Omnibus tests of model coefficients at 5% level of significance **(Figure 1)**.

Dissemination of results/findings

Is this necessary? pls format according to Journal's publication format

Findings of this research was submitted and presented to Aksum University, college of health sciences for the partial fulfillment of master of public health nutrition. It will also be disseminated to both hospitals. I will make an attempt to submit it for publication in one reputable journal as well.

Results

Out of total 585 eligible severely malnourished children hospitalized to the NRUs of public hospitals in Aksum, 564 children were followed from September 11, 2016 to February 10, 2019 with retrieval rate of 96.4%. Out of the total followed children, 56% were recovered from SAM and recovery times for the remaining 44% were considered to be censored at the

time of analysis. The recovery time analysis was made on the basis of demographics and various health related characteristics of study subjects.

Socio-demographic characteristics, co-morbidity and type of SAM

More than half (53.9%) of the children enrolled in to the study were males with the corresponding median recovery time of 15 days (95% CI: 13.7, 16.3), similar with the overall median LOS of 15 days (95% CI: 14.1, 15.9).

Age was grouped into categories in convenience for analysis. Median survival time was measured in days by sex and age categories of the study participants. The largest percentage of children was categorized under the age group of 12-35 months which accounted for 54.6% of the total subjects with an estimated median recovery time of 15 days in a 95% CI (13.7, 16.3). In contrast, the percentage of children under age group 0-5 months were only 10.8 %, the least in percentage comparison despite its association with the highest median recovery time of 20 days with a 95% CI (16.4, 23.6).

The median recovery time for children with Marasmus which accounted for 64.9% of the study participants was found to be 15 days with 95% CI (13.8, 16.2), the same point estimate with those having Kwashiorkor (20.7 % of the total). The remaining 14.4% were children with Marasmic-Kwashiorkor having, 17 days of average length of recovery time (95% CI; 13.6, 20.4) **(Table 2**).

At admission, pneumonia was the most commonly observed co-morbidity (38.6%) followed by anemia (16.2%) and TB (9.4%). The presence of co-morbidity contributed to the delay in recovery. It was observed that co-morbid children (64.4%) had an average LOS of 17 days with 95 % CI (15.8, 18.2). This observed difference in duration of recovery was also supported by the log rank test, and hence, co-morbidity as a potential factor was checked

further and found to be significant predictor of LOS. However, longer recovery time associated with Marasmic-Kwashiorkor was only due to chance that was not significantly different from duration of recovery of children with Marasmus and Kwashiorkor at 5% level of significance. Median survival time calculated in days by type of SAM and presence or absence of co-morbidity **(Table 2).**

Clinical conditions of patients at admission

Most of the children (98%) were newly admitted and majority of them were in severe condition at admission. Seventy one point one and fifty eight point five per cent of children come up with a complaint of diarrhea and vomiting respectively while 45.2% of them had deranged pulse rate (PR), 67.2% had dehydration (18.8%, 46.6% and 34.6% were with mild, moderate and severe dehydration respectively) and 37.4% showed up with palmar pallor. 41.8% of study participants had altered consciousness level (39.5% lethargic and 2.3% comatose), 21.8 % had skin lesion while 11.5 and 14.5 percent of the children were febrile and hypothermic respectively. Among the total participants, 3.2 and 49.8% had reactive and non-reactive sero-status respectively. Among edematous children, bilateral type of edema was most common (90.5%) and grade three (+++) edema was commonly observed in 42.6% of edematous subjects whereas the remaining 41 and 16.4 per cent were grade two (++) and one (+) respectively.

During Bivariate analysis the presence of diarrhea, vomiting, fever, hypothermia, malaria, HIV, edema, palmar pallor, skin lesion, dehydration, and shock at admission were all suggestive of significantly related with longer duration of recovery than their absence and

were selected for multivariate analysis. However, the observed differences in average recovery time due to the presence of malaria and edema were not found significant according to the log rank test and hence, were not further tested for predicting recovery time in Cox regression multivariate analysis.

Treatment, treatment outcomes, routine medications and feedings

Out of the participants enrolled, 56% were discharged cured, 22.2% transferred to nearby health facility, 9.8% defaulted, 6% dead during treatment and 6% were medical referral. 18.3 And 8 per cent of participants received IV fluid and blood transfusion respectively. In Bivariate analysis, not receiving both IV fluid and blood transfusion found significantly associated with shorter LOS till recovery and both were selected for multivariate analysis to check if predictors of LOS to recover from SAM.

Almost all (98.8%) received antibiotics like amoxicillin, ampicillin and gentamycin and higher numbers of children were supplemented folic acid and iron (68.1% and 61.9% respectively). Regarding feedings, F75 was offered for almost all patients (99.8%) followed by F100 (for 81% of participants) and half of them were given plumpy nut. Meanwhile, 31.9 per cent were fed with NG tube. As receiving folic acid, vitamin A, F100, plumpy nut and not being fed with NG tube were suggestive of shorter recovery time in bivariate analysis, these all were selected for further multivariable analysis using Cox regression model **(Table 3)**. For those discharged cured, the average weight gain was 10.1g/kg/day. The overall median recovery time from SAM was also determined to be 15 days with a 95% confidence interval between 14.1 and 15.9 days **(Figure 2)**.

Predictors of length of stay

After ascertaining validity of the model assumptions and adjustment, eight independent significant predictors of LOS for nutritional recovery were found, which are; diarrhea, HIV sero status, palmar pallor, co-morbidity, blood transfusion, IV fluid infusion, provision of plumpy nut and feeding with the help of NG tube.

Patients with diarrhea at admission were 42.7% (AHR = 0.573; 95% CI: 0.415-0.793) less likely to recover quickly from SAM as compared to those without diarrhea while those HIV positive found to be 60.9% (AHR = 0.391; 95% CI: 0.194-0.788) less likely to get cured fast in comparison with those whose sero-status is unknown. However, no significant difference was obtained in the hazards of non-recovery for children with Non-reactive (AHR = 0.937; 95% CI: 0.714-1.231) and unknown HIV status. The difference in chance of recovery between reactive and non-reactive patients stays significant at 5% level of significance.

Children with palmar pallor and co-morbidity at admission were 42.5% (AHR = 0.575; 95% CI: 0.416-0.794) and 58.5% (AHR = 0.415; 95% CI: 0.302-0.570) less likely to recover earlier as compared to those who did not have such conditions while patients who were not fed with NG tube and not infused with IV fluid were 1.9 times (AHR = 1.861; 95% CI: 1.335-2.593) and 1.55 times (AHR = 1.548; 95% CI: 1.074-2.232) more likely to recover fast in reference to those fed with NG tube and infused with IV fluid respectively.

Patients who were not treated with plumpy nut were 63.2% (AHR = 0.368; 95% CI: 0.262-0.518) less likely to recover in comparison with those who received plumpy nut. Unsurprisingly, the highest difference in median LOS was observed in patients who were not

transfused, 1.9 times (AHR = 1.905; 95% CI: 1.158-3.135) more likely to recover than their counter parts who were transfused.

Discussion

This research was conducted on 564 under five years of age children admitted to the NRUs of the two public hospitals in Aksum town, reviewed retrospectively from September 11, 2016 to February 10, 2019 to assess LOS till recovery from SAM and associated factors. Results of this study were examined for consistency with extensive review of literatures of the title.

At admission, 64.4% of participants showed up with other co-morbidities on top of SAM. Though it shows less amount of children were co-morbid compared to findings from Bahirdar and Debrebrhan (1,31), it is compatible with the reports from Bahirdar, Gedeo and Jimma (32,35,37). Majority of children (64.9%) were hospitalized with marasmus (non-edematous) type of SAM, similar with some recent studies in Ethiopia (1,13,31,32,36,40–43) but in contrary to that of Jimma (37), Hadiya (44) and Uganda (30), which reported edematous type of SAM was highly encountered. This variation might be attributable to the multi-faceted causes of SAM all over the world.

In this enquiry, the median LOS till recovery from SAM was estimated to be 15 days (95% CI: 14.1, 15.9), which is in the acceptable range of international standards set by the SPHERE project (15). This is similar with the findings from institution based researches in Bahirdar (13,33) that reported 16 days of recovery time. But this finding is far better than the report from Sidama zone shebedino district of southern Ethiopia (41) which found LOS of 36

This is not the way a scientific discussion should be

days. It is also better (lower LOS) than multiple studies in the country (1,8,11,35,37) and than that of Yemen (28). However, it is longer than some study reports from some parts of Ethiopia (31,32,44), Ghana (20) and India (26). This could be due to the differences in underlying co-morbidities, caring practice of healthcare providers, health facility set up and variation in socioeconomic status of the population in these different study areas. In this study, as it also was in recent studies in Ethiopia (40,42,43), type of SAM was not associated with statistically significant difference in recovery time among edematous and severely wasted children, in contrary to the current finding from southern region of Ethiopia which shows marasmic ones were less likely to recover earlier compared to their edematous counter parts (41).

The overall rate of recovery from SAM was found to be 56%, that is consistent with findings from Debrebrhan University that revealed 55.9% rate of recovery (31). It is better than that of Ayder hospital and other similar studies from Bahirdar (11,13,33), and far better than the findings from Ghana and Yemen (20,28). Nevertheless, it is by greater margin below the minimum international standards (15), in comparison with other study findings in Ethiopia (1,8,32,35–37,40–43) and similar reports from India, Malawi and Uganda (25,26,29,30) as well. This might be due to the relatively higher rate of transfer out to nearby health facility, which probably would be to prevent patient overload since one of the study area (AkUCSH) is the only referral hospital in this particular study area. Twenty two point two per cent of patients were transferred out to nearby health facility that is above other recently reported study findings in Ethiopia (1,13,32,33,36), Ghana (20), Uganda (30) and Yemen (28).

This study shows death rate of 6% from the total participants enrolled, which is acceptable by the SPHERE project minimum international standards for managing SAM in NRU/TFU, and

better than the recent study findings from University of Gondar (40) and Hawassa University (43) comprehensive specialized hospitals which found mortality rates of 10.8 % for each. It is similar with the findings of two studies done in Ethiopia (31,32) and one conducted in Nigeria (38). But the mortality rate is greater than that of some studies done in Ethiopia (11,13,33), India (25,26), Ghana (20) and Yemen (28). This could be due to lack of close follow up of patients with strict adherence to the national or international SAM management protocols and socioeconomic differences in the different areas.

The average weight gain in this thesis was in line with the international/national set of standards, unlike the recent finding from southern Ethiopia, Sidama zone, that found an average weight gain of 5.4 g/kg/day (41). This variation could be due to the study setting, as the latter was conducted in OTP (which exclude complicated SAM), that found 35.2 and 20.8% of sharing and selling the home taken plumpy nut respectively.

According to the multivariate analysis, there was significant difference in median LOS till cured from SAM among predictor variables. Participants who showed up with palmar pallor (indicative of anemia) at admission were by 42.5 per cent (AHR = 0.575; 95% CI: 0.416-0.794) less likely to have fast recovery than those who did not have pallor. This is in line with the findings reported from Nekemte and Bahirdar felegehiwot referral hospitals (10,13). Similarly, not receiving plumpy nut (AHR = 0.368; 95% CI: 0.262-0.518) was observed as strong independent predictor of recovery time in this thesis, which also is consistent with report from Bahirdar (13). But other related studies from Ethiopia (9,32) reported that neither palmar pallor nor plumpy nut as independent predictor of LOS till recovery. This could be due to inter institutional differences in strictly adhering to the national SAM management guideline.

In line with this research findings, studies from different parts of the country (1,10,32) and studies from Malawi and Uganda (29,30) reported that children presented with retroviral infection at hospitalization were less likely to recover from SAM, as being reactive for HIV serostatus among the study participants had negatively affected LOS to recover from SAM (AHR = 0.391; 95% CI: 0.194-0.788) in this particular thesis work.

Probability of getting cured fast was reduced by 58.5% (AHR = 0.415; 95% CI: 0.302-0.570) in those admitted with co-morbidity. Consistent with this report, a hospital based retrospective cross-sectional study from Bahirdar and retrospective cohort study in similar setting in Jimma University found less recovery rate of SAM in co-morbid children (1,37). Another finding from this research is that those not fed with NG tube had 1.9 times (AHR = 1.861; 95% CI: 1.335-2.593) higher chance of faster recovery in reference to those fed with NG tube, that is in agreement with study finding in Gedeo zone of southern Ethiopia (35).

Limitations of the study

Even though the strength of this thesis paper comes from its study design (cohort), it totally was based on patients' secondary data, in which incompleteness was observed to some extent, and lacked control over the quality of measurements taken during hospitalization. It was also impossible to analyze socio-economic characteristics of parents/guardians and factors related to patient treatment (medical/pharmaceutical supplies and healthcare provider expertise) that could have influenced the outcome variable in a desirable or undesirable way.

Conclusion

This research figured out that the median LOS till recovery is in the acceptable range of the national and international standards set to manage SAM as in patient (7,15). However, the rate of recovery was lower as compared to the stated standards and other study findings conducted in the nation.

It also revealed significant differences in the median LOS to recover among different predictor variables. Using multivariate Cox proportional hazard regression model, the study proved that children that showed up with presence of co-morbidity, diarrhea, palmar pallor and retroviral infection, and those who did not receive plumpy nut had lower chance of recovering early. Likewise, children who did not blood transfused, resuscitated with IV fluid and not fed with the help of NG tube had greater chance of getting cured as compared to their counter parts that were transfused, resuscitated with IV fluid and NG tube fed. Moreover, the management team of both hospitals along with zonal health office and regional health bureau are strongly expected to supervise and mentor the medical staff involved in the NRUs to assess if complying with the national SAM management protocols and fill gaps accordingly.

Meanwhile, staffs assigned to the nutrition rehabilitation wards are required to be vigilant enough to strictly follow the inpatient SAM management guidelines endorsed by the federal ministry of health in treating patients coming with diarrhea, pallor (anemia), HIV/AIDS, and providing plumpy nut on the proper time.

Finally, to enrich and compensate the limitations of this research, the scholars in Aksum University are strongly advised to conduct further study prospectively so that it can be triangulated (explore perceptions of parents/guardians about SAM and the services given).

Declarations

Ethical approval

Ethical clearance and permission letter were taken from the Ethical Review Committee (ERC) of Aksum University, College of health science. An official letter was written to Selekleka primary hospital requesting facilitation to conduct the research study and this official letter approved and distributed to the respective units of Selekleka primary hospital. Confidentiality and privacy was maintained during data collection, analysis and reporting in which the information obtained from the data will not be shared other than the data collectors and principal investigator and this was assured by obtaining institutional written consent from Selekleka primary hospital and by providing Information sheet which explains the purpose, benefit, and short and long-term effect of the study on the study participants.

Consent for publication

Not applicable

Availability of data and materials

The datasets used and/or analyzed during the current study are available from the corresponding author on reasonable request.

Competing interests

No competing interests.

Funding

No funding was obtained for this study

Authors' contributions

Conceptualization: WT

Data curation: WT, MA, SH, TZ

Formal analysis: WT, MA, SH, Tz

Funding acquisition: WT

Investigation: WT

Methodology: WT, MA, SH

Project admiration: WT

Resources: WT, MA, SH, TZ

Software: WT, MA, SH, TZ

Supervision: MA, SH, TZ

Validation: MA, SH

Visualization: WT, MA, SH, TZ

Writing original draft: TZ

Writing review and editing: TZ, TA, MA, KT

Acknowledgements

I am cordially thankful for the data collectors, the supervisor and staffs of St. Mary general hospital and Aksum University comprehensive specialized hospital for their support during data collection.

References

1. Desyibelew HD, Fekadu A, Woldie H. Recovery rate and associated factors of children age 6 to 59 months admitted with severe acute malnutrition at inpatient unit of Bahir Dar Felege Hiwot Referral hospital therapeutic feeding unite , northwest Ethiopia. 2017;18:1–12.
2. Collins S, Dent N, Binns P, Bahwere P, Sadler K, Hallam A. Review Management of severe acute malnutrition in children; 2006;
3. Williams PCM, Berkley PJA. SAM update : current WHO guidelines and WHO essential medicine list for children; 2016.
4. Medecins Sans frontieres (MSF). Nutrition guidelines, 2nd edition; May. 2006.
5. Gerensea H, Berhe T, Siyum H. Journal of Anesthesia & Clinical Trend of Malnutrition in Tigray Region from 2011/12-2014/15. 2014;8(5):8–11.
6. World health organization (WHO). Management of severe malnutrition : a manual for

physicians and other senior health workers; 1999.

7. Federal ministry of health (FMoH). Training course on the management of severe acute malnutrition, Federal democratic Republic of Ethiopia , Ministry of Health; 2013;

8. Mengesha MM, Deyessa N, Sileshi B. Treatment outcome and factors affecting time to recovery in children with severe acute malnutrition treated at outpatient therapeutic care program Treatment outcome and factors affecting time to recovery in children with severe acute malnutrition treated at outpatient therapeutic care program. 2016;(July).

9. Gebremichael DY. Predictors of nutritional recovery time and survival status among children with severe acute malnutrition who have been managed in therapeutic feeding centers , Southern Ethiopia : retrospective cohort study. BMC Public Health [Internet]. 2015;1–11. Available from: http://dx.doi.org/10.1186/s12889-015-2593-5

10. Mena MB, Dedefo MG. Treatment Outcome of Severe Acute Malnutrition and Its Determinants among Pediatric Patients in West Ethiopia; 2018;

11. Tirore MG, Atey TM, Mezgebe HB. Survival status and factors associated with treatment outcome of severely malnourished children admitted to Ayder referral hospital : a cross-sectional study; 2017;1–9.

12. UNICEF/WHO/World bank. Levels and trends in child malnutrition. 2018;1–16.

13. Asres DT, Prasad RPCJ, Ayele TA. Recovery time and associated factors of severe acute malnutrition among children in Bahir Dar city , Northwest Ethiopia : an

institution based retrospective cohort study; 2018;1–8.

14. Ashworth A, Schofield C. Guidelines for the inpatient treatment of severely malnourished children.

15. The Sphere project. Humanitarian charter and minimum standards in humanitarian response; 2011.

16. Trehan I, Manary MJ. Management of severe acute malnutrition in low-income and middle-income countries; 2015;283–7.

17. Gachau S, Irimu G, Ayieko P, Akech S, Agweyu A. Prevalence , outcome and quality of care among children hospitalized with severe acute malnutrition in Kenyan hospitals : A multi-site observational study; 2018;1–17.

18. Besada D, Kerber K, Leon N, Sanders D, Daviaud E, Rohde S, et al. Niger ' s Child Survival Success , Contributing Factors and Challenges to Sustainability : A Retrospective Analysis; 2016;1–18.

19. Awoke et al. BMC Nutrition. Determinants of severe acute malnutrition among under five children in rural Enebsie. 2018;1–8.

20. Saaka M, Osman SM, Amponsem A, Ziem JB, Abdul-mumin A, Akanbong P, et al. Treatment Outcome of Severe Acute Malnutrition Cases at the Tamale Teaching Hospital; 2015;

21. UNICEF. Evaluation of community management of acute malnutrition (cmam); Ethiopia country casestudy. 2012;(September).

22. Federal Democratic Republic of Ethiopia. Ethiopian demographic health survey. 2016.

23. Tigray RHB. The Government of the National Regional State of Tigray, Bureau of Health. Trends of Key health Indicator in Tigray; 2016.

24. Aprameya HS, Kamath SP, Kini PK, Baliga BS, Shenoy U V, Jain A. Socioepidemiological determinants of severe acute malnutrition and effectiveness of nutritional rehabilitation center in its management. 2015;148–53.

25. Das S, Paul DK, Bhattacharya M, Basu S, Chatterjee A, Sen S. Clinicoepidemiological Profile , Risk Factors and Outcome of Severe Acute Malnutrition Children at the Nutritional Rehabilitation Centre of a Tertiary Care Centre in Eastern India- A 4 Years Experience. 2017;5(2):2–9.

26. Tiwari AK, Jaiswal AK, Saurabh K. A study of clinical profile and outcome of SAM children admitted in nutritional rehabilitation centre , Patna Medical College and Hospital ,. 2018;5(4):1497–504.

27. Rusmil VK, Padjadjaran U. Factors Influencing Outcomes of Children Hospitalized with Acute Severe Malnutrition Factors Influencing Outcomes of Children Hospitalized with Acute Severe Malnutrition. 2018;(October).

28. Muftah S. Outpatient management of severe acute malnutrition among children under five years old , in Yemen : a retrospective cohort study; 2016;3(2):445–51.

29. Trehan I, Goldbach HS, Lagrone LN, Meuli GJ, Wang RJ, Maleta KM, et al. Antibiotics as part of the management of severe acute malnutrition. 2016;28(September).

30. Nyeko R, Calbi V, Ssegujja BO, Ayot GF. Treatment outcome among children under-five years hospitalized with severe acute malnutrition in St . Mary ' s hospital Lacor , Northern Uganda. BMC Nutr [Internet]. 2016;1–7. Available from: http://dx.doi.org/10.1186/s40795-016-0058-6

31. Derseh B, Mruts K, Demie T, Gebremariam T. Co-morbidity , treatment outcomes and factors affecting the recovery rate of under -five children with severe acute malnutrition admitted in selected hospitals from Ethiopia : retrospective follow up study. 2018;1–8.

32. Mekuria G, Derese T, Hailu G. Treatment outcome and associated factors of severe acute malnutrition among 6 – 59 months old children in Debre Markos and Finote Selam hospitals , Northwest Ethiopia : a retrospective cohort study. 2017;1–8.

33. Desalegn Gebrezgi, Degnet Teferi PPR. Recovery time from severe acute malnutrition and development of complementary food supplement for affected Ethiopian children; 2019;(3):1–6.

34. Desta KS. Nutritional Disorders & Therapy Survival Status and Predictors of Mortality among Children Aged 0-59 Months with Severe Acute Malnutrition Admitted to Stabilization Center at Sekota Hospital Waghemra Zone. 2015;5(2).

35. Tadele Girum EM and AW. Comparative Analysis of the Survival Status and Treatment Outcome of Under-five Children Admitted with Severe Acute Malnutrition Among Hospital-based and Health Center Based Stabilization Abstract : 2018;209–20.

36. Kabeta A, Bekele G. Factors Associated with Treatment Outcomes of Under-five

Children with Severe Acute Malnutrition Admitted to Therapeutic Feeding Unit of Yirgalem Hospital Clinics in Mother and Child Health. 2017;14(2).

37. Jarso H, Workicho A, Alemseged F. Survival status and predictors of mortality in severely malnourished children admitted to Jimma University Specialized Hospital from 2010 to 2012 , Jimma , Ethiopia : a retrospective longitudinal study. BMC Pediatr [Internet]. 2015;1–13. Available from: http://dx.doi.org/10.1186/s12887-015-0398-4

38. John C, Diala U, Adah R, Lar L, Envuladu EA, Adedeji I, et al. Survival and nutritional status of children with severe acute malnutrition , six months post-discharge from outpatient treatment in Jigawa state , Nigeria; 2018;64:1–10.

39. Mwanza M, Okop KJ, Puoane T. Evaluation of outpatient therapeutic programme for management of severe acute malnutrition in three districts of the eastern province , Zambia. BMC Nutr [Internet]. 2016;1–9. Available from: http://dx.doi.org/10.1186/s40795-016-0102-6

40. Wagnew F, Dejenu G, Eshetie S, Alebel A, Worku W. Treatment cure rate and its predictors among children with severe acute malnutrition in northwest Ethiopia : A retrospective record review. 2019;288:1–13.

41. Teshome G, Bosha T, Gebremedhin S. Time-to-recovery from severe acute malnutrition in children 6 – 59 months of age enrolled in the outpatient treatment program in Shebedino , Southern Ethiopia : a prospective cohort study. 2019;1–10.

42. Hassen SL, Astatkie A, Mekonnen TC, Bogale GG. Survival Status and Its

Determinants among Under-Five Children with Severe Acute Malnutrition Admitted to Inpatient Therapeutic Feeding Centers in South Wollo Zone , Amhara Region , Ethiopia. 2019;

43. Fikrie A, Alemayehu A, Gebremedhin S. Treatment outcomes and factors affecting time-to-recovery from severe acute malnutrition in 6 – 59 months old children admitted to a stabilization center in Southern Ethiopia : A retrospective cohort study. 2019;1–9.

44. Yohannes T, Laelago T, Ayele M, Tamrat T. Mortality and morbidity trends and predictors of mortality in under-five children with severe acute malnutrition in Hadiya zone , South Ethiopia : a four-year retrospective review of hospital-based. 2017;1–8.

Table 1: Sample size calculation

Study	P	AHR	Margin of error (α)	Power of study	Rate of incompleteness	Sample size
Jimma University (Jarso et al. 2015)	22.2% (0.222)	1.9 (age < 24 Months)	0.05	80%	10%	378
		3.0(Hypothermia presence)	0.05	80%	10%	130
		2.6(impaired consciousness)	0.05	80%	10%	171
Wolkite University (Girum et al. 2018)	17.1% (0.171)	2.44 (altered pulse rate)	0.05	80%	10%	254
		1.8 (NG tube insertion)	0.05	80%	10%	585
		2.78 (Hypoglycemia present)	0.05	80%	10%	194

Where P = Prevalence of non-recovery (at third week)

Table 1: Log Rank (Mantel-Cox) test of equality of survival distribution for socio-demographic characteristics, type of SAM and co-morbidity at admission in severely malnourished children admitted to public hospitals in Aksum, 2019

Variable	Category	Percentage of outcome category						Median Survival (days)	95% CI		Log-rank test	
		Cured %	Dead %	Defaulted %	Transferred %	Referred %	Total %		LL	UL	Chi-square	P-value
Sex	Male	31.4	2.3	4.8	12.2	3.2	53.9	15	13.7	16.3	2.87	0.090
	Female	24.6	3.7	5.0	9.9	2.8	46.1	16	14.6	17.4		
Age (in months)	0-5	7.3	1.1	0.7	1.1	0.7	10.8	20	16.4	23.6	8.74	0.033
	6-11	10.1	0.5	2.1	5.5	1.6	19.9	14	13.2	14.8		
	12-35	29.8	3.4	5.5	12.9	3.0	54.6	15	13.7	16.3		
	36-59	8.9	1.1	1.4	2.7	0.7	14.7	15	13.1	16.9		
Name of institution	AkUCSH	23.2	2.3	4.6	6.0	3.9	40.1	16	14.1	17.9	7.34	0.007
	St. Mary hospital	32.8	3.7	5.1	16.1	2.1	59.9	15	13.9	16.1		
Type of SAM	Marasmus	35.5	3.5	6.9	14.9	4.1	64.9	15	13.8	16.2	0.384	0.825
	Kwashiorkor	11.2	1.1	2.0	5.1	1.4	20.7	15	13.1	16.9		
	Marasmic-kwash	9.4	1.4	0.9	2.1	0.5	14.4	17	13.6	20.4		
Co-morbidity	Present	38.8	6.0	5.0	8.7	5.9	64.4	17	15.8	18.2	82.82	<0.001
	Absent	17.2	0.0	4.8	13.5	0.2	35.6	11	10.0	12.0		
Total		56.0	6.0	9.8	22.2	6.0	100.0	15	14.1	15.9		

 Click here to access/download;Figure;Table 3.docx

Table 1: Log Rank (Mantel-Cox) test of equality of survival distribution for clinical conditions at admission, treatment and routine medications in severely malnourished children admitted to public hospitals, Aksum, 2019

Variable	Category	Percentage of outcome category						Median survival (days)	95% CI		Log rank test	
		Cured %	Dead %	Defaulted %	Transferred %	Referred %	Total %		LL	UL	Chi-square	P-value
Diarrhea	Present	41.7	5.7	6.7	12.9	4.1	71.1	16	14.7	17.3	28.74	<0.001
	Absent	14.4	0.4	3.1	9.2	2.0	28.9	13	11.5	14.5		
Vomiting	Present	33.5	5.1	5.2	11	3.7	58.5	17	15.6	18.4	13.38	<0.001
	Absent	22.5	0.9	4.6	11.2	2.3	41.5	14	13.1	14.9		
Fever	Present	5.7	2.0	0.7	1.3	2.0	11.5	19	15.6	22.4	5.41	0.02
	Absent	50.4	4.0	9.1	20.9	4.0	88.5	15	14.0	16.0		
Hypothermia	Present	7.6	3.0	1.2	1.6	1.0	14.5	21	15.6	26.4	17.46	<0.001
	Absent	48.4	3.0	8.6	20.6	5.0	85.5	15	14.1	15.9		
Malaria	Present	2.1	0.0	0.0	0.2	0.0	2.3	16	14.4	17.6	0.78	0.377
	Absent	53.9	6.0	9.8	22.0	6.0	97.7	15	14.0	16.0		
HIV status	Reactive	2.3	0.9	0.0	0.0	0.0	3.2	28	19.4	36.6	14.20	0.001
	Non-reactive	30.9	3.4	3.4	8.7	3.5	49.8	15	13.5	16.5		
	Unknown	22.9	1.8	6.4	13.5	2.5	47.0	15	13.9	16.1		
Pulse rate	Bradycardic	0.5	0.4	0.0	0.0	0.4	1.3	13	5.0	21.0	27.56	<0.001
	Normal	30.0	0.6	6.5	13.8	2.8	53.8	14	12.9	15.1		
	Tachycardic	25.5	5.1	3.3	8.4	2.8	44.9	18	16	20.0		
Consciousness level	Conscious	33.0	0.4	6.6	16.5	1.8	58.2				42.597	<0.001
	Lethargic	23.0	3.9	3.0	5.5	4.0	39.5					
	Comatose	0.0	1.8	0.2	0.2	0.2	2.3					
Edema	Present	21.1	2.5	2.9	7.3	1.8	35.5	16	14.5	17.5	0.05	0.827
	Absent	34.9	3.5	6.9	14.9	4.2	64.5	15	13.8	16.2		
Palmar pallor	Present	21.6	4.6	2.4	5.0	3.9	37.4	19	17.4	20.6	39.60	<0.001
	Absent	34.4	1.4	7.4	17.2	2.1	62.6	14	13.2	14.8		
Skin lesion	Present	14.5	2.8	0.6	2.5	1.4	21.8	20	16.4	23.6	22.04	<0.001
	Absent	41.5	3.2	9.2	19.7	4.6	78.2	14	13.1	14.9		
Dehydration	Present	41	6.0	6.7	9.3	4.3	67.2	16	14.7	17.3	35.90	<0.001
	Absent	15	0.0	3.1	12.9	1.8	32.8	12	10.7	13.3		

Table 3: Survival distributions for clinical conditions at admission, treatment and routine medications, Aksum, 2019 (Continued)

Variable	Category	Percentage of outcome category						Median survival (days)	95% CI		Log rank test	
		Cured %	Dead %	Defaulted %	Transferred %	Referred %	Total %		LL	UL	Chi-square	P-value
Shock	No	55.0	4.0	9.6	22.0	5.7	96.3	15	14.1	16.0	16.87	<0.001
	Yes	1.0	2.0	0.2	0.2	0.4	3.7	33	21.3	44.7		
Transfusion	No	51.8	3.2	9.8	22.2	5.1	92.0	14	13.1	14.9	25.92	<0.001
	Yes	4.2	2.8	0.0	0.0	0.9	8.0	27	18.9	35.1		
IV infusion	No	43.6	3.2	9.4	20.2	5.3	81.7	14	13.4	14.7	24.69	<0.001
	Yes	12.4	2.8	0.4	2.0	0.7	18.3	20	18.5	21.5		
Amox/ampicillin/ gentamycin	No	0.5	0.2	0.2	0.4	0.0	1.2	12	4.7	19.3	2.07	0.150
	Yes	55.5	5.8	9.6	21.8	6.0	98.8	15	14.1	15.9		
Anti malaria treatment	No	54.0	5.7	9.8	22.0	6.0	97.5	15	14	16	0.72	0.395
	Yes	2.0	0.4	0.0	0.2	0.0	2.5	15	12.9	17.1		
Deworming	No	29.6	4.6	8.7	13.7	5.3	61.9	16	14.8	17.2	0.04	0.852
	Yes	26.4	1.4	1.1	8.5	0.7	38.1	15	13.5	16.6		
Measles vaccinated	No	38.5	5.8	8.5	16.2	6.0	75.1	15	14	16.1	0.47	0.492
	Yes	17.5	0.2	1.3	6.0	0.0	24.9	15	13.1	16.9		
Folic acid	No	12.2	3.4	6.1	8.2	2.1	31.9	17	15.7	18.3	7.35	0.007
	Yes	43.8	2.7	3.7	14	3.9	68.1	14	12.9	15.1		
Vitamin A	No	13.5	4.6	6.1	5.5	3.4	33	18	16.1	19.9	12.93	<0.001
	Yes	42.5	1.4	3.7	16.7	2.6	67	14	13.1	14.9		
Iron	No	14.9	4.3	7.4	9.8	1.8	38.1	15	13.1	16.9	1.08	0.300
	Yes	41.1	1.7	2.4	12.4	4.2	61.9	15	13.9	16.1		
F-75	No	0.0	0.0	0.0	0.0	0.2	0.2				0.04	0.845
	Yes	56.0	6.0	9.8	22.2	5.8	99.8					
F-100	No	4.3	3.2	5.1	3.2	3.2	19.0	20	14.2	25.8	5.89	0.015
	Yes	51.7	2.8	4.7	19.0	2.8	81.0	15	14.1	16		
Plumpy nut	No	9.9	6.0	9.8	18.8	5.5	50.0	21	17.1	24.9	55.44	<0.001
	Yes	46.1	0.0	0.0	3.4	0.5	50.0	14	13.3	14.7		
NG Tube feeding	No	36.3	0.4	9.1	20.1	2.3	68.1	13	12.3	13.7	102.2	<0.001
	Yes	19.7	5.6	0.7	2.1	3.7	31.9	21	18.5	23.5		
Total		56.0	6.0	9.8	22.2	6.0	100	15	14.1	15.9		

Table 1: Factors associated with LOS to recover from SAM in children admitted to public hospitals in Aksum, 2019

Variable	Category	Recovery status				CHR (95% CI)	P-value	AHR (95% CI)
		Non-recovered		Recovered				
		Number	Percent	Number	Percent			
Sex	Male	127	22.5	177	31.4	1.204(0.963, 1.505)	0.104	
	Female	121	21.5	139	24.6	1		
Age	0-5	20	3.5	41	7.3	0.639(0.421, 0.969)	0.048*	0.702(0.369, 1.334)
	6-11	55	9.8	57	10.1	1.105(0.755, 1.616)		1.102(0.711, 1.706)
	12-35	140	24.8	168	29.8	0.982(0.715, 1.348)		1.012(0.717, 1.428)
	36-59	33	5.9	50	8.9	1		1
Name of institution	AkUCSH	95	16.8	131	23.2	1.354(1.077, 1.702)	0.01*	0.775(0.573, 1.048)
	St. Mary hospital	153	27.1	185	32.8	1		1
Type of SAM	Marasmus	166	29.4	200	35.5	0.974(0.844, 1.124)	0.717	
	Kwash	54	9.6	63	11.2			
	Mixed	28	5.0	53	9.4	1		
Co-morbidity	Present	144	25.5	219	38.8	3.103(2.384, 4.039)	**<0.001****	0.415(0.302, 0.570)
	Absent	104	18.4	97	17.2	1		1
Diarrhea	Present	166	29.4	235	41.7	1.973(1.518, 2.565)	**<0.001****	0.573(0.415, 0.793)
	Absent	82	14.5	81	14.4	1		1
Vomiting	Present	141	25.0	189	33.5	1.509(1.198, 1.901)	<0.001*	0.883(0.684, 1.138)
	Absent	107	19.0	127	22.5	1		1
Fever	Present	33	5.9	32	5.7	1.515(1.049, 2.187)	0.027*	1.066(0.695, 1.637)
	Absent	215	38.1	284	50.4	1		1
Hypothermia	Present	39	6.9	43	7.6	1.929(1.391, 2.674)	<0.001*	0.767(0.529, 1.112)
	Absent	209	37.1	273	48.4	1		1
Malaria	Present	1	0.2	12	2.1	0.779(0.436, 1.390)	0.398	
	Absent	247	43.8	304	53.9	1		
HIV status	Reactive	5	0.9	13	2.3	1.314(1.091, 1.583)	**0.004****	0.391(0.194, 0.788)
	Non-reactive	107	19.0	174	30.9			0.937(0.714, 1.231)
	Unknown	136	24.1	129	22.9	1		1

Table 4: Factors associated with LOS to recover from SAM, public hospitals in Aksum, 2019 (Continued)

Variable	Category	Recovery status				CHR (95% CI)	P-value	AHR (95% CI)
		Non-recovered		Recovered				
		Number	Percent	Number	Percent			
Pulse rate	Bradycardic	4	0.8	3	0.6	0.559(0.445, 0.702)	<0.001*	2.227(0.676, 7.336)
	Normal	124	23.5	160	30.3			1.363(1.054, 1.762)
	Tachycardic	101	19.1	136	25.8	1		1
Consciousness level	Conscious	142	25.2	186	33.0	0.492(0.393, 0.616)	<0.001*	14.29(0.00, 93.70)
	Lethargic	93	16.5	130	23.0			11.53(0.00, 75.59)
	Comatose	13	2.3	0	0.0	1		1
Edema	Present	81	14.4	119	21.1	1.025(0.816, 1.287)	0.834	
	Absent	167	29.6	197	34.9	1		
Palmar pallor	Present	89	15.8	122	21.6	2.036(1.610, 2.574)	**<0.001****	0.575(0.416, 0.794)
	Absent	159	28.2	194	34.4	1		1
Skin lesion	Present	41	7.3	82	14.5	1.80(1.389, 2.332)	<0.001*	0.982(0.697, 1.384)
	Absent	207	36.7	234	41.5	1		1
Dehydration	Present	148	26.2	231	41	2.143(1.645, 2.791)	<0.001*	1.107(0.802, 1.528)
	Absent	100	17.7	85	15.1	1		1
Shock	No	233	41.3	310	55	0.196(0.080, 0.481)	<0.001*	1.481(0.584, 3.755)
	Yes	15	2.7	6	1.1	1		1
Transfusion	No	227	40.2	292	51.8	0.364(0.239, 0.555)	**<0.001****	1.905(1.158, 3.135)
	Yes	21	3.7	24	4.3	1		1
IV infusion	No	215	38.1	246	43.6	0.521(0.397, 0.684)	**<0.001****	1.548(1.074, 2.232)
	Yes	33	5.9	70	12.4	1		1
Amox/ampicil lin/gentamycin	No	4	0.7	3	0.5	0.452(0.144, 1.417)	0.173	
	Yes	244	43.3	313	55.5	1		
Anti malaria treatment	No	245	43.5	305	54.0	1.285(0.703, 2.351)	0.415	
	Yes	3	0.5	11	2.0	1		
Deworming	No	182	32.3	167	29.6	1.021(0.817, 1.274)	0.857	
	Yes	66	11.7	149	26.4	1		
Measles vaccinated	No	206	36.6	217	38.5	1.084(0.853, 1.377)	0.510	
	Yes	41	7.3	99	17.6	1		

Table 4: Factors associated with LOS to recover from SAM, public hospitals in Aksum, 2019 (Continued)

Variable	Category	Recovery status				CHR (95% CI)	P-value	AHR (95% CI)
		Non-recovered		Recovered				
		Number	Percent	Number	Percent			
Folic acid	No	111	19.7	69	12.2	1.427(1.090, 1.868)	0.010*	1.003(0.670, 1.502)
	Yes	137	24.3	247	43.8	1		1
Vitamin A	No	110	19.5	76	13.5	1.575(1.214, 2.043)	0.001*	0.881(0.635, 1.223)
	Yes	138	24.5	240	42.6	1		1
Iron	No	131	23.2	84	14.9	1.136(0.884, 1.459)	0.319	
	Yes	117	20.8	232	41.1	1		
F-75	No	1	0.2	0	0.0	20.149(0.000, 101)	0.897	
	Yes	247	43.8	316	56.0	1		
F-100	No	83	14.7	24	4.3	1.639(1.077, 2.493)	0.021*	1.284(0.779, 2.117)
	Yes	165	29.3	292	51.7	1		1
Plumpy nut	No	226	40.1	56	9.9	2.774(2.073, 3.711)	**<0.001****	0.368(0.262, 0.518)
	Yes	22	3.9	260	46.1	1		1
NG Tube feeding	No	179	31.7	205	36.3	0.287(0.221, 0.374)	**<0.001****	1.861(1.335, 2.593)
	Yes	69	12.2	111	19.7	1		1
Total		248	44.0	316	56.0			

NB: * = significant during bivariate analysis and selected for multivariate Cox regression, **** =** significant predictors of length of stay (LOS)

Click here to access/download;Figure;Table 5.docx

Table 1: Performance indicators of TFUs in public hospitals, Aksum, 2019

Indicators	Achieved	SPHERE project reference values	
		Acceptable	Alarming
Recovery rate	56.0 %	> 75 %	< 50 %
Death rate	6.0 %	< 10 %	> 15 %
Defaulter	9.8 %	< 15 %	> 25 %
LOS in days	15 days	< 28 days	> 42 days
Weight gain in g/kg/day	10.1 g/kg/day	> = 8 g/kg/day	< = 5 g/kg/day

Figure Click here to access/download;Figure;Figure 1.docx

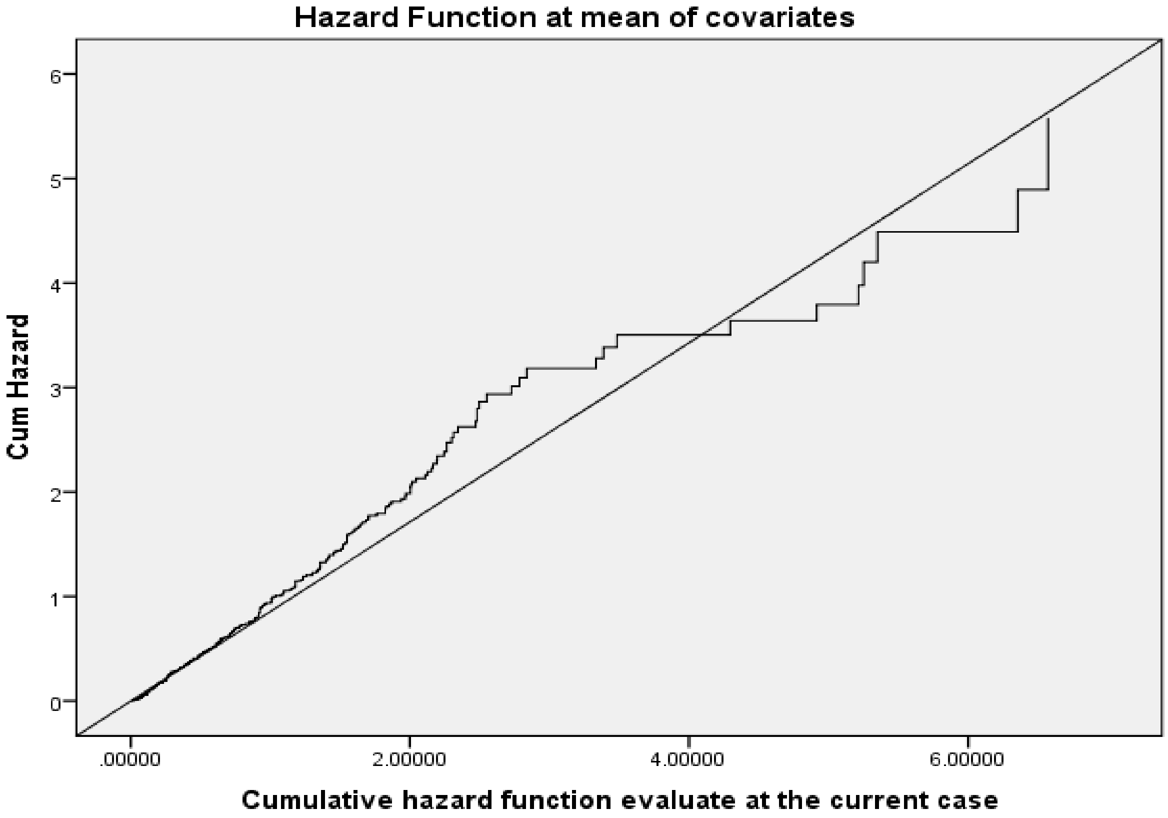

Figure 1: Cumulative hazard plots of Cox Snell Residual

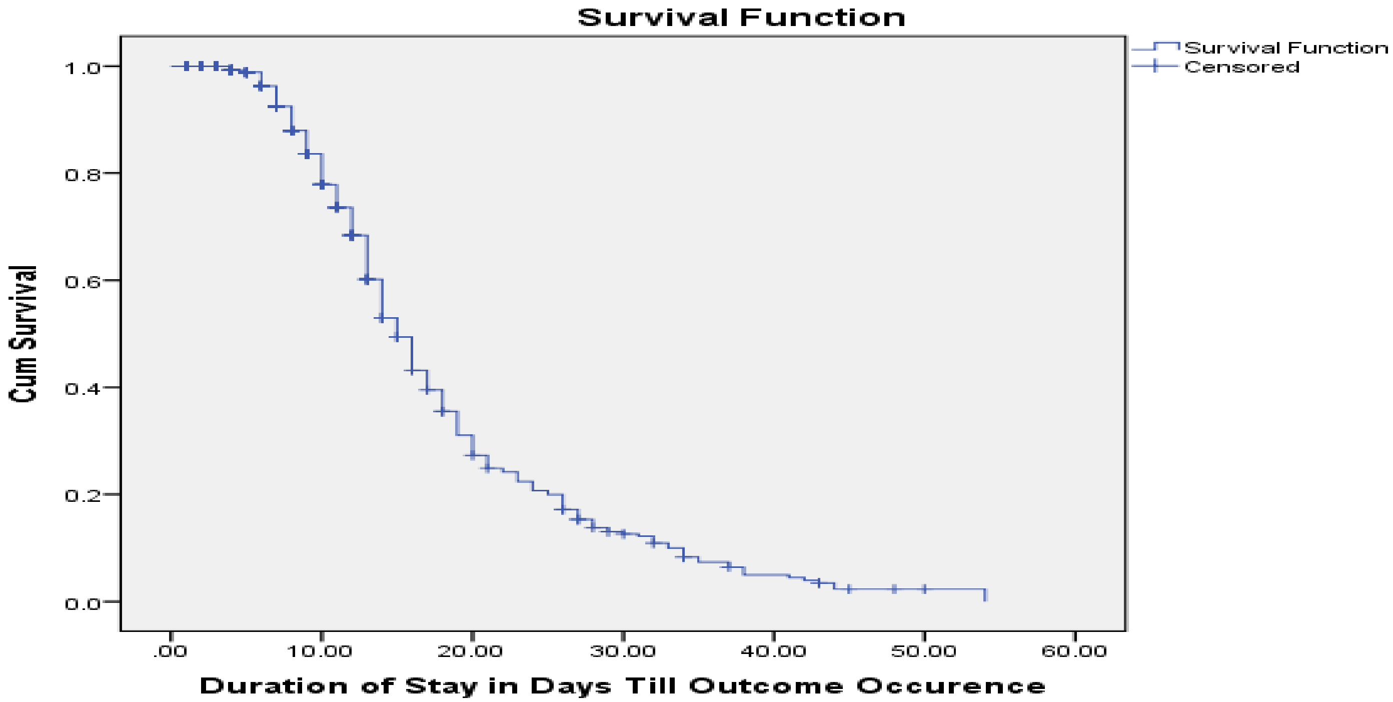

Figure 2: Overall survival function of children with SAM admitted to public hospitals in Aksum, 2019

Figure
Click here to access/download;Figure;Figure 3.docx

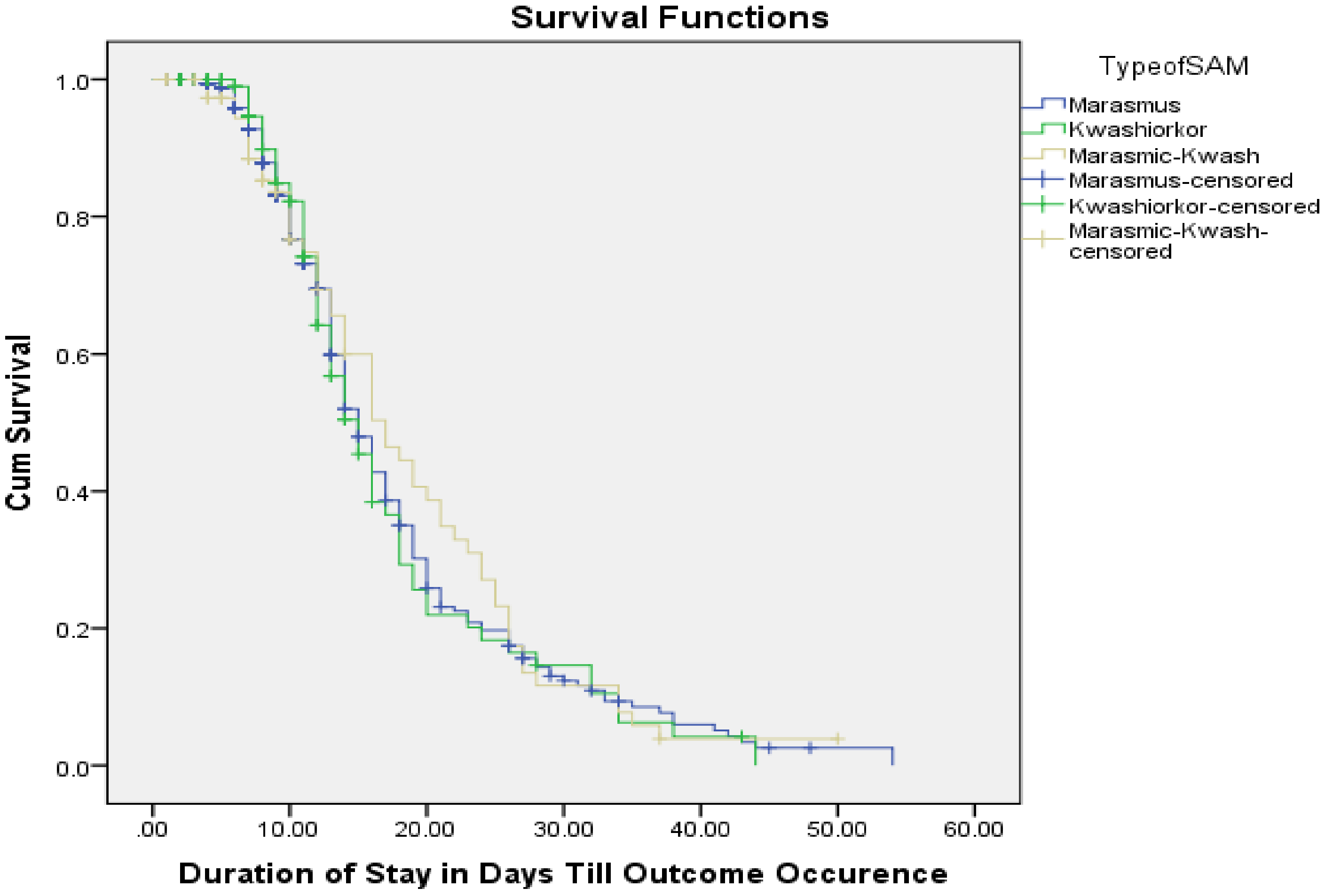

Figure 3: Survival function by type of SAM, Aksum, 2019
