# Supplementary material for: Length of stay to recover from severe acute malnutrition and associated factors among under-five years children admitted to public hospitals in Aksum, Ethiopia
Source: PLoS One. 2020 Sep 29;15(9):e0238311. doi: 10.1371/journal.pone.0238311 (PMC7523984; doi:10.1371/journal.pone.0238311)
Supplement: Attachment — Submitted filename: Response to Reviewers.docx [file pone.0238311.s003.docx]

Response to Reviewers

**Reviewer #1**

| **S.N** | **Reviewer’s Concern/Questions** | **Response** | **Found in**  **(Line number)** |
| --- | --- | --- | --- |
| 1. | Some of the words/sentences used clearly reflects that this was taken directly from a thesis without careful revision by all the authors. This is ridiculous. | Corrected | Throughout the manuscript |
| 2. | Although the study has revealed significant differences in the median LOS to recover among different predictor variables nevertheless, in general the findings are highlighting that the length of stay is in the acceptable range of the international and national set of standards. In addition the findings are more relevant to Hospitals in Aksum and not sure if the findings will be impactful/relevant to other regions of Africa /countries experiencing SAM children. Probably this should be highlighted further | We tried to include all public hospitals of the town. As we can see in the discussion section the finding of the length of stay and its determinants are more or less similar with other parts of the country and other countries which have similar socio-demographic and economic status. | Line 288-323 |
| 3. | Some methodology parts are included in the background section of the manuscript | Corrected | Line 15-23 |
| 4. | Redundancy of the objective of the study (at the end of the introduction section) | Omitted based on your recommendation | Line 104-106 |
| 5. | Mis-inclusion of dissemination of the result (At the end of the methods section)-not the standard of PLOS one | Omitted based on PLoS ONE Journal Requirement | Check the last paragraph of the methods section  (starting from line #207) |
| 6. | Typographic errors | Corrected | Throughout the manuscript |
| 7. | Non-standard way of writing the discussion section | Modified accordingly |  |

Response to Reviewers

**Reviewer #2**

| **S.N** | **Reviewer’s Concern/Questions** | **Response** | **Found in**  **(Line number)** |
| --- | --- | --- | --- |
| 1. | The aim as stated (line106) “this research was intended to come up with information about length of stay till recovery and associated factors among severely malnourished children admitted to the identified health facilities for the betterment of quality of care.” It was however, not clear whether the intention was to see “the effect of associated factors on the length of stay in the hospital” or “to find out what were the associated factors in children who were admitted with severe acute malnutrition”. Of course, on reading the research paper it becomes clear that the objective is to see the effect. | The main intention of the second objective was to identify independent predictors/factors for the length of stay (short/long) to recover from SAM. | Line 106 |
| 2. | On page 17 under predictors of length of stay: “five independent significant predictors of LOS for nutritional recovery were found, which are; diarrhea, HIV sero status, palmar pallor (anemia), co-morbidity, provision of plumpy nut and feeding with the help of NG tube”. I would think diarrhea, HIV sero-status, palmar pallor should be included under “co-morbidity” and thus “co-morbidity” per se would not be an independent predictor of the length of stay. | As indicated in other similar studies, HIV sero-status, Diarrheal disease, Anemia are found the common determinants. In our study, based on the previous findings, we need to give more emphasis to these factors as independent predictors of the length of stay. The other-comorbidities (TB, Malaria, Pneumonia, consists of comorbidities other than the common predictors mentioned above. | - Line 175 of the methods, - Line 258 of the result, and - Footnote of Table 2 |
| 3. | Similarly, “deranged pulse rate”, “severe dehydration,” “altered consciousness”, “shock” and “hypothermia” under the heading “Clinical conditions of patients at admission (page 15, lines 295-308)” would all probably be parts of the same clinical spectrum at admission. These are all analyzed separately for incidence. | We found that analyzing the incidence to recovery of each of these many clinical features of the severely malnourished children is irrelevant and not in lined with the objective of the study. Hence we totally omitted these from the manuscript. |  |
| 4. | Early/quick recovery has not been defined (Line 337 & 344-45). | Right comment and corrected.  This is now changed to recovered and not recovered, as the outcome variable is a binary outcome | Result section  (Line 256-270) |
| 5. | “Probability of getting cured fast was reduced by 58.5% (AHR = 0.415; 95% CI: 0.302-0.570) in those admitted with co-morbidity” (Line 424). Are the terms “recovery” and “cure” used interchangeably here? | Yes, we now change the term cure to recovery | Line 266 (result) |
| 6. | Some abbreviations are not explained, e.g. “OTP (line96) and NRU (205) which I presume is the therapy unit where the children were admitted. | Clarified in detail | Line 92-93 |
| 7. | I thought the references should include the names of journals where they are originally published. They are of course easily traceable. | Corrected, We now use Vancouver method of referencing using an EndNote software. | See the list of references (Line 380) |
